# Non-contiguous finished genome sequence and description of *Bacillus massiliogorillae* sp. nov.

**DOI:** 10.4056/sigs.4388124

**Published:** 2013-10-02

**Authors:** Mamadou Bhoye Keita, Seydina M. Diene, Catherine Robert, Didier Raoult, Pierre-Edouard Fournier, Fadi Bittar

**Affiliations:** 1 URMITE, Aix-Marseille Université, Faculté de Médecine, Marseille, France; 2 King Fahad Medical Research Center, King Abdul Aziz University, Jeddah, Saudi Arabia

**Keywords:** *Bacillus massiliogorillae*, genome, culturomics, taxonogenomics

## Abstract

Strain G2^T^ sp. nov. is the type strain of *B. massiliogorillae*, a proposed new species within the genus *Bacillus*. This strain, whose genome is described here, was isolated in France from the fecal sample of a wild western lowland gorilla from Cameroon. *B. massiliogorillae* is a facultative anaerobic, Gram-variable, rod-shaped bacterium. Here we describe the features of this organism, together with the complete genome sequence and annotation. The 5,431,633 bp long genome (1 chromosome but no plasmid) contains 5,179 protein-coding and 98 RNA genes, including 91 tRNA genes.

## Introduction

Strain G2^T^ (= CSUR P206 = DSM 26159) is the type strain of *B. massiliogorillae* sp. nov. This bacterium is a Gram-variable, facultatively anaerobic, indole-negative bacillus having rounded-ends. It was isolated from the stool sample of *Gorilla gorilla gorilla* as part of a “culturomics” study aiming at cultivating bacterial species within gorilla feces.

The genus *Bacillus* (Cohn 1872) was created about 140 years ago [1]. To date this genus, comprised mostly of Gram-positive, motile, and spore-forming bacteria, includes 276 species with validly published names [2]. Members of the genus *Bacillus* are ubiquitous bacteria isolated from various environments including soil, fresh and sea water, food, and occasionally from humans and animals in which they are either pathogens, such as *B. anthracis* (the causative agent of anthrax) [3] and *B. cereus* (associated mainly with food poisoning) [4], or saprophytes [5]. *Bacillus* species may also rarely be involved in a variety of human infections, including pneumonia, bacteremia, meningitis, endocarditis, endophthalmitis, osteomyelitis and skin/soft tissue infection [5]. However, in great apes, few data are available about the presence of the genus *Bacillus*. Recent reports have described the isolation of atypical *B. anthracis* (*B. anthracis*-like bacteria) in wild chimpanzees and gorillas from Africa [6-8].

Here we present a summary classification and a set of features for *B. massiliogorillae* sp. nov. strain G2^T^ together with the description of the complete genome sequence and annotation. These characteristics support the circumscription of the species *B. massiliogorillae* [9].

## Classification and features

**Figure 1:**
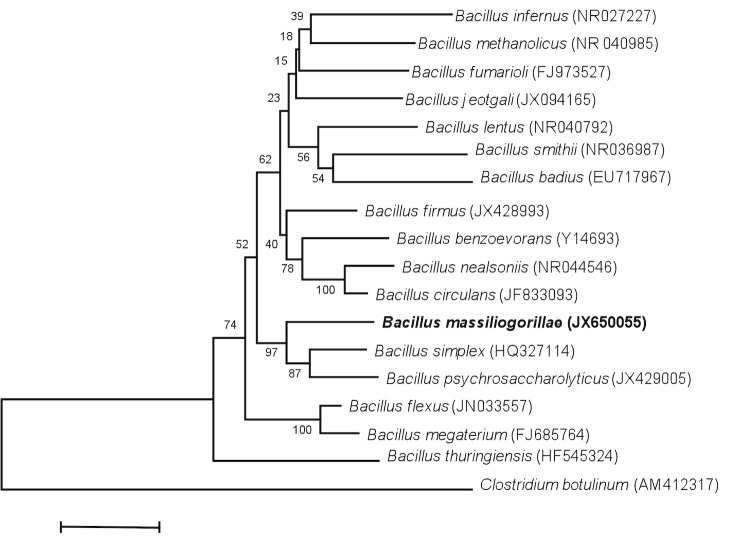
Phylogenetic tree highlighting the position of *Bacillus massiliogorillae* strain G2^T^ relative to other type strains within the *Bacillus* genus. GenBank accession numbers are indicated in parentheses. Sequences were aligned using CLUSTAL X (V2), and phylogenetic inferences obtained using the maximum-likelihood method within the MEGA 5 software [22]. Numbers at the nodes are percentages of bootstrap values obtained by repeating the analysis 1,000 times to generate a majority consensus tree. *Clostridium botulinum* was used as outgroup. The scale bar represents a 2% nucleotide sequence divergence.

In July 2011, a fecal sample was collected from a wild western lowland gorilla near Messok, a village in the south-eastern part of the DJA FAUNAL Park (Cameroon). The collection of the stool sample was approved by the Ministry of Scientific Research and Innovation of Cameroon. No experimentation was conducted on this gorilla. The fecal specimen was preserved at -80°C after collection and sent to Marseille. Strain G2^T^ (Table 1) was isolated in January 2012 by cultivation on *Brucella* agar medium (Oxoid, Dardilly, France). This strain exhibited a 97.3% 16S rRNA nucleotide sequence similarity with *Bacillus simplex*, the phylogenetically closest validly published *Bacillus* species (Figure 1). This value was lower than the 98.7% 16S rRNA gene sequence threshold recommended by Stackebrandtia and Beers to delineate a new species without carrying out DNA-DNA hybridization [23].

**Table 1:** Classification and general features of *Bacillus massiliogorillae* strain G2^T^

**MIGS ID**	**Property**	**Term**	**Evidence code^a^**
	Current classification	Domain *Bacteria* Phylum *Firmicutes* Class *Bacilli* Order *Bacillales* Family *Bacillaceae* Genus *Bacillus* Species *Bacillus massiliogorillae* Type strain G2^T^	TAS [10] TAS [11-13] TAS [14,15] TAS [16,17] TAS [16,18] TAS [16,19,20] IDA IDA
	Gram stain	Variable	IDA
	Cell shape	Rod	IDA
	Motility	Motile	IDA
	Sporulation	Sporulating	IDA
	Temperature range	Mesophile	IDA
	Optimum temperature	37°C	IDA
MIGS-6.3	Salinity	Growth in BHI medium + 2% NaCl	IDA
MIGS-22	Oxygen requirement	Facultative anaerobic	IDA
	Carbon source	Unknown	NAS
	Energy source	Unknown	NAS
MIGS-6	Habitat	Gorilla gut	IDA
MIGS-15	Biotic relationship	Free living	IDA
MIGS-14	Pathogenicity Biosafety level Isolation	Unknown 2 Gorilla feces	NAS
MIGS-4	Geographic location	Cameroon	IDA
MIGS-5	Sample collection time	July 2011	IDA
MIGS-4.1	Latitude	Unknown	NAS
MIGS-4.1	Longitude	Unknown	NAS
MIGS-4.3	Depth	Unknown	NAS
MIGS-4.4	Altitude	Unknown	NAS

Evidence codes - IDA: Inferred from Direct Assay; TAS: Traceable Author Statement (i.e., a direct report exists in the literature); NAS: Non-traceable Author Statement (i.e., not directly observed for the living, isolated sample, but based on a generally accepted property for the species, or anecdotal evidence). These evidence codes are from the Gene Ontology project [21]. If the evidence is IDA, then the property was directly observed for a live isolate by one of the authors or an expert mentioned in the acknowledgements.

**Figure 2:**
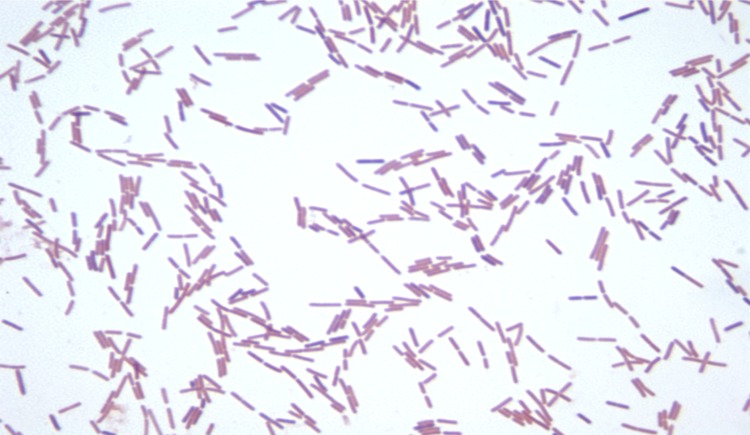
Gram staining of *B. massiliogorillae* strain G2^T^

**Figure 3:**
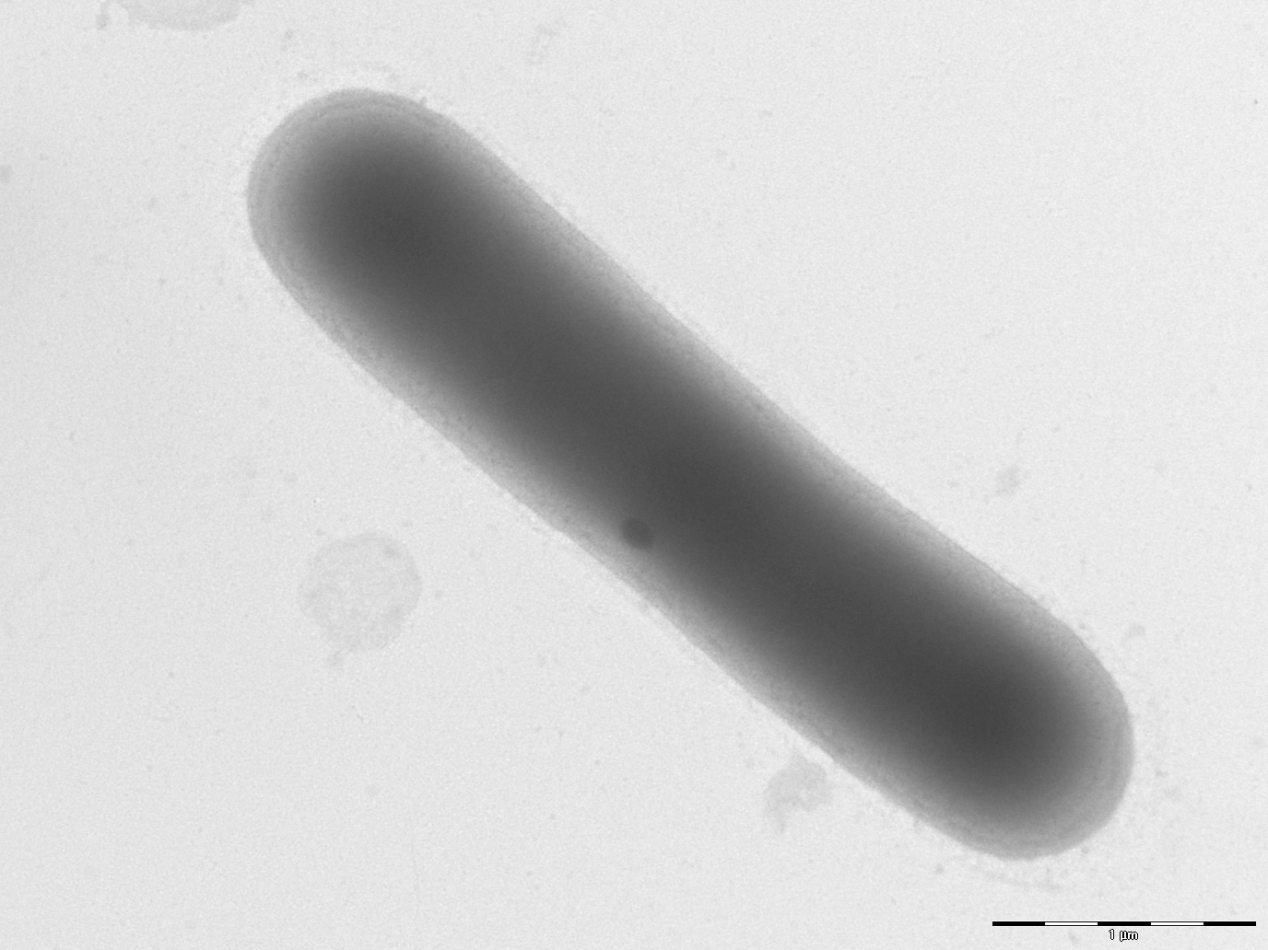
Transmission electron microscopy of *B. massiliogorillae* strain G2^T^, using a Morgani 268D (Philips) at an operating voltage of 60kV. The scale bar represents 1 µm.

Different growth temperatures (25, 30, 37, 45°C) were tested. Growth occurred at all tested temperatures, and the optimal growth was observed at 37°C. Colonies were 2-5 mm in diameter on Columbia agar, grey opaque in color. Growth of the strain was tested under anaerobic and microaerophilic conditions using GENbag anaer and GENbag microaer systems, respectively (BioMérieux), and in aerobic conditions, with or without 5% CO_2_. Growth was achieved under aerobic (with and without CO_2_), microaerophilic and anaerobic conditions. Gram staining showed Gram variable bacilli (Figure 2). A motility test was positive. Cells grown on agar sporulate and the rods have a length ranging from 3.2 to 7.5 µm (mean 5.4 µm) and a diameter ranging from 0.8 to 1.2 µm (mean 1 µm) as determined by negative staining transmission electron microscopy (Figure 3).

Strain G2^T^ exhibited catalase activity but not oxidase activity. Using the API 50CH system (BioMerieux), a positive reaction was observed for D-glucose, D-fructose, D-ribose, N-acetylglucosamine, amygdalin, arbutin, aesculin, salicin, cellobiose, maltose, D-lactose, D-trehalose, D-saccharose, and hydrolysis of starch. Using the API ZYM system, positive reactions were observed for esterase (C4), esterase lipase (C8), phosphatase acid, α- glucosidase and N-acetyl-β-glucosaminidase. The urease reaction was also positive, but nitrate reduction and indole production were negative. *B. massiliogorillae* is susceptible to amoxicillin, nitrofurantoin, erythromycin, doxycycline, rifampin, vancomycin, gentamycin and imipenem but resistant to trimethoprim- sulfamethoxazole, ciprofloxacin, ceftriaxon and amoxicillin-clavulanic acid.

When compared to other *Bacillus* species, *B. massiliogorillae* differed from *B. simplex* [24] for the utilization of amygdalin, cellobiose, lactose and glucose (Table 2). It also differed from *B. psychrosaccharolyticus* [25] in nitrate reductase and β-galactosidase production, and in the utilization of L-arabinose, mannitol, xylose and glycerol (Table 2). Differences were also observed with *B. circulans* [26] in β-galactosidase production and the utilization of D-mannose, L-arabinose, D-xylose, mannitol, arabinose, xylose, glycerol and D-galactose (Table 2).

**Table 2:** Differential phenotypic characteristics between *B. massiliogorillae* sp. nov. strain G2^T^ and phylogenetically close *Bacillus* species.

**Characteristic**	*B. massiliogorillae* sp. nov.	*B. simplex*	*B. psychrosaccharolyticus*	*B. circulans*
Cell diameter (µm)	0.87-1.2	0.7-0.9	0.9-1	0.5-0.8
Oxygen requirement	aerobic	aerobic	facultative anaerobic	aerobic
Gram stain	var	var	var	var
Salt requirement	< 5%	<7%	<10%	<7%
Motility	+	v	+	+
Endospore formation	+	+	+	+
				
**Production of**				
Alkaline phosphatase	+	na	na	na
Acid phosphatase	+	na	na	na
Catalase	+	+	+	+
Oxidase	-	-	na	na
Nitrate reductase	-	na	+	na
Urease	+	na	na	w
α-galactosidase	-	na	na	na
β- galactosidase	-	na	+	+
β-glucuronidase	-	na	na	na
α -glucosidase	+	na	na	na
N-acetyl- β -glucosaminidase	+	na	na	na
Indole	-	na	na	na
Esterase	+	na	na	na
Esterase lipase	+	na	na	na
Naphthyl-AS-BI-phosphohydrolase	-	na	na	na
Phenylalanine arylamidase	-	na	na	na
Leucine arylamidase	-	na	na	na
Cystine arylamidase	-	na	na	na
Valine arylamidase	-	na	na	na
Glycine arylamidase	-	na	na	na
				
**Utilization of**				
D-mannose	-	-	na	+
Amygdalin	+	-	na	v
L-Arabinose	-	-	+	+
Cellobiose	+	-	na	+
Lactose	+	-	+	+
D-xylose	-	-/w	na	+
Glucose	+	na	+	+
Mannitol	-	na	+	+
Arabinose	-	na	+	+
Xylose	-	na	+	+
Glycerol	-	na	+	+
D-Galactose	-	na	na	+
Starch	+	na	na	+
				
**Habitat**	gorilla gut	soil	soil and lowland marshes	environment and fish gut

var: variable, +: positive result, -: negative result, na: data not available, w: weak positive result

**Figure 4:**
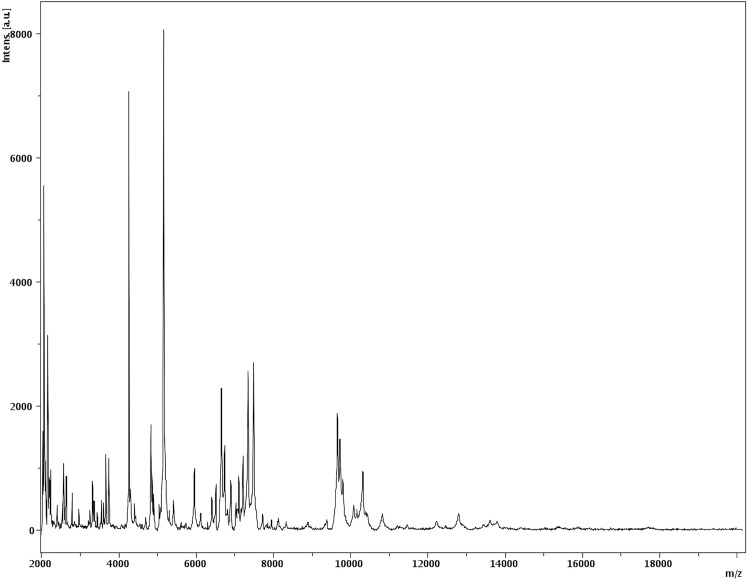
Reference mass spectrum from *B. massiliogorillae* strain G2^T^. Spectra from 12 individual colonies were compared and a reference spectrum was generated.

**Figure 5:**
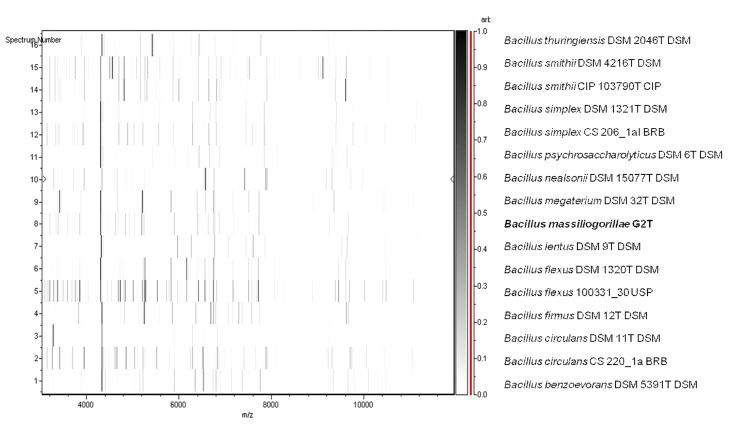
Gel view comparing *Bacillus massiliogorillae* G2^T^ spectra with other members of the *Bacillus* genus (*B. thuringiensis, B. smithii, B. simplex, B. psychrosaccharolyticus, B. nealsonii, B. megaterium, B. lentus, B. flexus, B. firmus, B. circulans* and *B. benzoevorans*). The Gel View displays the raw spectra of all loaded spectrum files arranged in a pseudo-gel like look. The x-axis records the m/z value. The left y-axis displays the running spectrum number originating from subsequent spectra loading. The peak intensity is expressed by a Gray scale scheme code. The color bar and the right y-axis indicate the relation between the color a peak is displayed with and the peak intensity in arbitrary units.

Matrix-assisted laser-desorption/ionization time-of-flight (MALDI-TOF) MS protein analysis was carried out as previously described [27,28]. Deposits were done for strain G2^T^ from 12 isolated colonies. Each smear was overlaid with 2µL of matrix solution (saturated solution of alpha-cyano-4-hydroxycinnamic acid) in 50% acetonitrile, 2.5% tri-fluoracetic-acid, and allowed to dry for five minutes. Measurements were performed with a Microflex spectrometer (Bruker Daltonics, Leipzig, Germany). Spectra were recorded in the positive linear mode for the mass range of 2,000 to 20,000 Da (parameter settings: ion source 1 (IS1), 20 kV; IS2, 18.5 kV; lens, 7 kV). A spectrum was obtained after 675 shots at a variable laser power. The time of acquisition was between 30 seconds and 1 minute per spot. The 12 G2^T^ spectra were imported into the MALDI BioTyper software (version 2.0, Bruker) and analyzed by standard pattern matching (with default parameter settings) against 6,252 bacterial spectra including 199 spectra from 104 *Bacillus* species, used as reference data, in the BioTyper database. The method of identification included the m/z from 3,000 to 15,000 Da. For every spectrum, 100 peaks at most were taken into account and compared with spectra in the database. A score enabled the identification, or not, from the tested species: a score > 2 with a validated species enabled the identification at the species level, a score > 1.7 but < 2 enabled the identification at the genus level; and a score < 1.7 did not enable any identification. For strain G2^T^, the scores obtained ranged from 1.177 to 1.343, thus suggesting that our isolate was not a member of a known species. We incremented our database with the spectrum from strain G2^T^ (Figure 4). Spectrum differences with other of *Bacillus* species are shown in Figure 5.

## Genome sequencing information

### Genome project history

The organism was selected for sequencing on the basis of its phylogenetic position and 16S rRNA similarity to other members of the genus *Bacillus*, and is part of a “culturomics” study of the gorilla flora aiming at isolating all bacterial species within gorilla feces. It was the 61^st^ genome of a *Bacillus* species and the first genome of *Bacillus massiliogorillae* sp. nov. A summary of the project information is shown in Table 2. The Genbank accession number is CAVL000000000 and consists of 66 large contigs. Table 3 shows the project information and its association with MIGS version 2.0 compliance [29].

**Table 3:** Project information

**MIGS ID**	**Property**	**Term**
MIGS-31	Finishing quality	High-quality draft
MIGS-28	Libraries used	454 paired-end 3- kb libraries
MIGS-29	Sequencing platform	454 GS FLX Titanium
MIGS-31.2	Sequencing coverage	13×
MIGS-30	Assemblers	Newbler version 2.5.3
MIGS-32	Gene calling method	Prodigal
	EMBL Date of Release	April 18, 2013
	EMBL ID	CAVL000000000
MIGS-13	Project relevance	Study of the gorilla gut microbiome

### Growth conditions and DNA isolation

*B. massiliogorillae* sp. nov. strain G2^T^, CSUR P206, DSM 26159, was grown aerobically on 5% sheep blood-enriched Columbia agar at 37°C. Four petri dishes were spread and resuspended in 3x500µl of TE buffer and stored at 80°C. Then, 500µl of this suspension were thawed, centrifuged 3 minutes at 10,000 rpm and resuspended in 3x100µL of G2 buffer (EZ1 DNA Tissue kit, Qiagen). A first mechanical lysis was performed by glass powder on the Fastprep-24 device (Sample Preparation system, MP Biomedicals, USA) using 2x20 seconds cycles. DNA was then treated with 2.5µg/µL lysozyme (30 minutes at 37°C) and extracted using the BioRobot EZ1 Advanced XL (Qiagen). The DNA was then concentrated and purified using the Qiamp kit (Qiagen). The yield and the concentration was measured by the Quant-it Picogreen kit (Invitrogen) on the Genios Tecan fluorometer at 50ng/µl.

### Genome sequencing and assembly

The paired-end library was prepared with 5 µg of bacterial DNA using the DNA fragmentation on the Covaris S-Series (S1, S2) instrument (Woburn, Massachusetts, USA) with an enrichment size at 3-5-kb. The DNA fragmentation was visualized through the Agilent 2100 BioAnalyzer on a DNA labchip 7500. The library was constructed according to the 454 GS FLX Titanium paired-end protocol (Roche). Circularization and nebulization were performed and generated a pattern with an optimum at 500 bp. After PCR amplification through 15 cycles followed by double size selection, the single stranded paired-end library was quantified using the Quant-it Ribogreen kit (Invitrogen) on the Genios Tecan fluorometer at 339 pg/µL. The library concentration equivalence was calculated as 1.00E+08 molecules/µL. The library was stored at -20°C until further use.

The paired-end library was clonally amplified with 0.5 cpb and 1 cpb in 2 emPCR reactions with the GS Titanium SV emPCR Kit (Lib-L) v2 (Roche). The yield of the emPCR was 19.4%, slightly above the expected yield ranging from 5 to 20% recommended by the Roche procedure.

Approximately 790,000 beads for a ¼ region were loaded on the GS Titanium PicoTiterPlate PTP Kit 70x75 and sequenced with the GS FLX Titanium Sequencing Kit XLR70 (Roche). The run was performed overnight and then analyzed on the cluster through the gsRunBrowser and Newbler assembler (Roche). A total of 322,962 passed filter wells were obtained and generated 64.2 Mb of sequences with a length average of 310 bp. The passed filter sequences were assembled using Newbler with 90% identity and 40 bp as overlap. The final assembly identified 60 scaffolds generating a genome size of 4.6 Mb.

### Genome annotation

Open Reading Frames (ORFs) were predicted using Prodigal [30] with default parameters but the predicted ORFs were excluded if they spanned a sequencing gap region. The predicted bacterial protein sequences were searched against the GenBank database [31] and the Clusters of Orthologous Groups (COG) databases using BLASTP. The tRNAScanSE tool [32] was used to find tRNA genes, whereas ribosomal RNAs were found by using RNAmmer [33] and BLASTn against the GenBank database. ORFans were identified if their BLASTP *E*-value was lower than 1e-03 for alignment length greater than 80 amino acids. If alignment lengths were smaller than 80 amino acids, we used an *E*-value of 1e-05.

To estimate the mean level of nucleotide sequence similarity at the genome level between *B. massiliogorillae* sp nov. strain G2^T^ and another 3 *Bacillus* species (Table 6), we compared genomes pairwise and determined the mean percentage of nucleotide sequence identity among orthologous ORFs using BLASTn. Orthologous genes were detected using the Proteinortho software [34].

**Table 6:** The number of orthologous proteins shared between genomes^†^

	*B. massiliogorillae*	*B. psychrosaccharolyticus*	*B. megaterium*	*B. thuringiensis*
*B. massiliogorillae*	**5,179**	70.15	69.28	69.66
*B. psychrosaccharolyticus*	1,936	**4,832**	68.74	68.46
*B. megaterium*	1,966	1,962	**5,100**	69.86
*B. thuringiensis*	1,877	1,873	1,903	**6,243**

^†^Lower left triangle- shared orthologous, upper right triangle- average percentage similarity of nucleotides corresponding to orthologous proteins shared between genomes, bold- number of proteins per genome

## Genome properties

**Figure 6:**
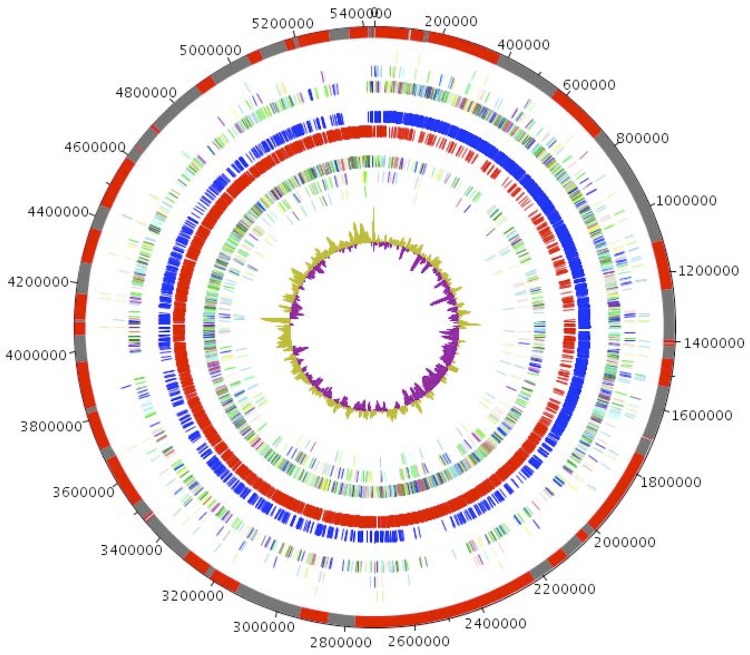
Graphical circular map of the genome. From outside in: contigs (red / grey), COG category of genes on the forward strand (three circles), genes on forward strand (blue circle), genes on the reverse strand (red circle), COG category on the reverse strand (three circles), GC content. The inner-most circle shows GC skew, purple and olive indicating negative and positive values, respectively.

The genome is 5,431,633 bp long (1 chromosome, but no plasmid) with a 34.95% G+C content (Figure 6 and Table 5). It is composed of 66 large contigs. Of the 5,276 predicted genes, 5,179 were protein-coding genes and 98 were RNAs (1 16S rRNA, 1 23S rRNA gene, 5 5S rRNA genes and 91 tRNA genes). A total of 3,801 genes (73.39%) were assigned a putative function (by COGS or by NR BLAST) and 368 genes were identified as ORFans (7.11%). The remaining genes were annotated as hypothetical proteins (666 genes, 12.86%). The distribution of genes into COGs functional categories is presented in Table 6. The properties and statistics of the genome are summarized in Tables 4 and 5.

**Table 5:** Number of genes associated with the 25 general COG functional categories

**Code**	**Value**	**% age**^a^	**Description**
J	180	3.48	Translation, ribosomal structure and biogenesis
A	0	0	RNA processing and modification
K	438	8.46	Transcription
L	191	3.69	Replication, recombination and repair
B	2	0.04	Chromatin structure and dynamics
D	42	0.81	Cell cycle control, mitosis and meiosis
Y	0	0	Nuclear structure
V	110	2.12	Defense mechanisms
T	275	5.31	Signal transduction mechanisms
M	182	3.51	Cell wall/membrane biogenesis
N	88	1.7	Cell motility
Z	0	0	Cytoskeleton
W	0	0	Extracellular structures
U	63	1.22	Intracellular trafficking and secretion
O	130	2.51	Posttranslational modification, protein turnover, chaperones
C	293	5.66	Energy production and conversion
G	247	4.77	Carbohydrate transport and metabolism
E	474	9.15	Amino acid transport and metabolism
F	110	2.12	Nucleotide transport and metabolism
H	177	3.42	Coenzyme transport and metabolism
I	188	3.63	Lipid transport and metabolism
P	300	5.79	Inorganic ion transport and metabolism
Q	133	2.57	Secondary metabolites biosynthesis, transport and catabolism
R	664	12.82	General function prediction only
S	344	6.64	Function unknown
-	1,269	24.50	Not in COGs

^a^ The total is based on the total number of protein coding genes in the annotated genome

**Table 4:** Nucleotide content and gene count levels of the genome

**Attribute**	**Value**	**% of total^a^**
Genome size (bp)	5,431,633	100
Coding region (bp)	4,561,287	83.98
G+C content (bp)	1,898,498	34.95
Total genes	5,276	100
RNA genes	98	1.84
Protein-coding genes	5,179	98.63
Genes with function prediction	3,801	73.39
Genes assigned to COGs	3,910	75.49
Genes with peptide signals	610	11.78
Genes with transmembrane helices	1,347	26.01

^a^ The total is based on either the size of the genome in base pairs or the total number of protein coding genes in the annotated genome

## Comparison with other *Bacillus* species genomes

Here, we compared the genome of *B. massiliogorillae* strain G2^T^ with those of *B. psychrosaccharolyticus* strain ATCC 23296, *B. megaterium* strain DSM 319 and *B. thuringiensis* strain ATCC 10792 (Table 6). The draft genome of *B. massiliogorillae* is larger in size than those of *B. psychrosaccharolyticus* and *B. megaterium* (5.43 vs 4.59 and 5.1 Mb, respectively) and smaller in size than that of *B. thuringiensis* (5.43 vs 6.26 Mb). *B. massiliogorillae* has a lower G+C content than *B. psychrosaccharolyticus* (34.95% vs 38.8%) and *B. megaterium* (34.95% vs 38.1%) but slightly higher than that *B. thuringiensis* (34.95% vs 34.8%). The protein content of *B. massiliogorillae* is higher than those of *B. psychrosaccharolyticus* and *B. megaterium* (5,179 vs 4,832 and 5,100 respectively) and fewer than that of *B. thuringiensis* (5,179 vs 6,243) (Table 6). In addition, *B. massiliogorillae* shares 1,936, 1,966 and 1,877 orthologous genes with *B. psychrosaccharolyticus*, *B. megaterium* and *B. thuringiensis* respectively (Table 6). The nucleotide sequence identity of orthologous genes ranges from 68.46 to 70.15% among *Bacillus* species, and from 69.28 to 70.15% between *B. massiliogorillae* and other *Bacillus* species (Table 6), thus confirming its new species status. Table 6 summarizes the number of orthologous genes and the average percentage of nucleotide sequence identity between the different genomes studied.

## Conclusion

On the basis of phenotypic (Table 2), phylogenetic and genomic analyses (taxonogenomics) (Table 6), we formally propose the creation of *Bacillus massiliogorillae* sp. nov. that contains the strain G2^T^. This strain has been found in a stool sample collected from gorilla in Cameroon.

### Description of *Bacillus massiliogorillae* sp. nov.

*Bacillus massiliogorillae* (ma.sil.io.go.ril’ae. L. gen. masc. n. *massiliogorillae*, combination of Massilia, the Latin name of Marseille, where strain G2^T^ was isolated, and of Gorilla, the Latin name of the gorilla, from which the stool sample was obtained).

*B. massiliogorillae* is an aerobic Gram-variable bacterium. Optimal growth is achieved aerobically. No growth is observed in microaerophilic or anaerobic conditions. Growth occurs on axenic media between 25 and 45°C, with optimal growth observed at 37°C. Cells stain Gram-positive or negative, are rod-shaped, endospore-forming, motile and have a mean diameter of 1 µm (range 0.8 to 1.2 µm) and a mean length of 5.4 µm (range 3.2 to 7.5 µm). Colonies are grey opaque and 2-5 mm in diameter on blood-enriched BHI agar.

Catalase positive but oxidase negative. Using the API 50CH system (BioMerieux), a positive reaction is obtained for D-glucose, D-fructose, D-ribose, N-acetylglucosamin, amygdalin, arbutin, aesculin, salicin, cellobiose, maltose, D-lactose, D-trehalose, D-saccharose, and hydrolysis of starch. Using the API ZYM system, positive reactions are obtained for esterase (C4), esterase lipase (C8), phosphatase acid, α- glucosidase and N-acetyl-β-glucosaminidase. Using API 20NE, there are neither nitrate reduction nor indole production but urease reaction was positive. Susceptible to amoxicillin, nitrofurantoin, erythromycin, doxycycline, rifampin, vancomycin, gentamycin and imipenem but resistant to trimethoprim- sulfamethoxazole, ciprofloxacin, ceftriaxon and amoxicillin-clavulanic acid.

The G+C content of the genome is 34.95%. The 16S rRNA and genome sequences are deposited in GenBank under accession numbers JX650055 and CAVL00000000, respectively. The type strain G2^T^ (= CSUR P206 = DSM 26159) was isolated from the fecal flora of a *Gorilla gorilla gorilla* from Cameroon.
